# Metabolism, senescence, and natural products: new perspectives on wound healing in diabetes

**DOI:** 10.3389/fnut.2025.1746827

**Published:** 2026-01-07

**Authors:** Jingping Wu, Qifeng Yang, Hongbin Cheng, Huilan Zheng, Mingling Chen

**Affiliations:** 1 Department of Medical Cosmetology, Hospital of Chengdu University of Traditional Chinese Medicine, Chengdu, China; 2 Department of Dermatology, Hospital of Chengdu University of Traditional Chinese Medicine, Chengdu, China

**Keywords:** cellular senescence, diabetic wounds, metabolic disorders, natural products, senescence-associated secretory phenotype

## Abstract

Chronic diabetic wounds have become a major clinical challenge because of their difficulty in healing and high recurrence rate. This review proposes for the first time the theoretical framework of the “metabolism–senescence axis,” systematically elucidating the central role of cellular senescence in the mechanisms underlying the impaired healing of diabetic wounds. Research has indicated that systemic and local metabolic disorders caused by hyperglycemia and insulin resistance directly drive the senescence process of local wound cells through multiple mechanisms, including mitochondrial dysfunction, oxidative stress, and the accumulation of advanced glycation end products. The accumulated senescent cells further exacerbate inflammation, inhibit repair cell function, and disrupt angiogenesis through the secretion of the senescence-associated secretory phenotype (SASP), thereby forming a vicious cycle. In terms of therapeutic strategies, various interventions have been developed, such as the selective clearance of senescent cells, SASP functional regulation, and metabolic reprogramming of senescent cells. Among these, natural products exhibit unique and irreplaceable advantages because of their multicomponent and multitarget characteristics: they can directly affect senescent cells and the SASP, synergistically regulate core signaling pathways, and reprogram metabolism while modulating the local microenvironment. The systematic integration of traditional compound therapies provides a rich arsenal for targeting senescence in diabetic wound treatment. Concurrently, the application of innovative delivery systems, such as smart-responsive hydrogels and microneedles, has effectively overcome the clinical translation bottlenecks posed by the inherent physicochemical properties of natural products. With the deepening integration of multidisciplinary approaches, the therapeutic paradigm for diabetic wounds is shifting from traditional empirical models to precision medicine, opening new avenues to overcome the therapeutic impasse of diabetic chronic wounds and achieve functional tissue repair.

## 1 Introduction

The continuous increase in the global incidence of diabetes has made the prevention and treatment of chronic diabetic wounds a critical global health priority. Among these, diabetic foot ulcers (DFUs) and its accompanying infection (DFI), which are the most representative and serious clinical phenotypes, constitute the core of this challenge. Research statistics indicate that the per-person-year recurrence rate of DFUs is as high as 22.1%, with significant regional variations, constituting a staggering global epidemiological burden (1). These refractory wounds not only severely impair patients’ quality of life but are also the leading cause of nontraumatic amputations, imposing a substantial economic burden on health care systems worldwide.

For a long time, research in this field has focused primarily on three traditional pathogenic factors: vascular lesions, neuropathy, and infection. However, in clinical practice, even when these factors are controlled, many wounds still struggle to heal. Recent research has revealed that skin wound healing is essentially a dynamically coordinated process involving multiple biological stages, such as the inflammatory response, angiogenesis, matrix deposition, and cell recruitment (2). Chronic diabetic wounds, however, are in a persistent pathological state because of various mechanisms, including inflammatory imbalance, protease dysregulation, insufficient angiogenesis, and impaired stem cell function (3). Further studies have indicated that impaired healing of chronic wounds in the elderly involves the synergistic effects of multiple factors, such as sustained inflammation and oxidative stress, biofilm formation, stem cell dysfunction, cellular senescence, and impaired angiogenesis (4), demonstrating high pathological complexity. In recent years, the research perspective in this field has expanded to a more systematic cognitive framework. A systematic review (5) established an integrated framework for diabetic wound research, systematically elucidating a series of core pathological processes from hyperglycemia-induced excessive reactive oxygen species (ROS) and inflammatory imbalance to angiogenesis disorders. This finding bridges traditional therapies with emerging strategies by focusing on key molecular targets and laying a theoretical foundation for the comprehensive management of this disease.

In this context, the proposal of the “geroscience hypothesis” provides a breakthrough perspective for understanding this challenge. The hypothesis explicitly states that targeting the fundamental biological process of aging may simultaneously delay multiple age-related diseases, including diabetes complications (6). Research on type 2 diabetes mellitus (T2DM) has confirmed that cellular senescence is the critical bridge linking aging and diabetes. Senescent cells in target organs, such as adipose tissue and pancreatic β-cells, induce a series of pathological changes, including insulin resistance, through the senescence-associated secretory phenotype (SASP) (7). Recent cross-tissue transcriptomic studies have further improved this understanding, revealing the common pathological features of “excessive inflammation activation” and “metabolic stagnation” in DFUs (8). These studies also precisely dissected tissue-specific molecular networks in skin (imbalanced keratinocyte proliferation and senescence), adipose tissue (adipocyte dedifferentiation and protease activation), and muscle (fibrosis and mitochondrial suppression) while identifying the IL-17 signaling and PPAR pathways as core dysregulated pathways mediating cross-tissue pathological communication. This complex multifactor, multilevel interaction suggests that intervention strategies targeting single mechanisms are unlikely to achieve ideal outcomes, necessitating the development of more comprehensive and systemic therapeutic approaches.

On the basis of the above understanding, this review systematically elaborates on the core role of the “metabolism–senescence axis” in the process of diabetic wound healing, with a focus on analyzing the heterogeneity and functional duality of senescent cells, how metabolic disorders drive cellular senescence, and how natural products intervene in this axis through multitarget mechanisms in depth, providing a new theoretical framework and therapeutic strategies for the comprehensive management of chronic diabetic wounds.

## 2 Diabetic wound healing impairment and cellular senescence

### 2.1 Evidence of senescence: from clinical observations to systems biology

Clinical studies have demonstrated at multiple levels that cellular senescence is a prevalent pathological feature of diabetic wounds, particularly DFUs. At the systemic level, in postmenopausal women, T2DM, but not obesity alone, is associated with elevated expression of p16 (especially variants 1 + 5) and p21 in T cells, as well as increased levels of various SASP factors (e.g., GDF-15 and IL-8). Furthermore, high p16 expression in T cells is significantly correlated with reduced tibial cortical area and thickness (9). These findings not only clarify the independent effects of hyperglycemia and obesity on cellular senescence but also suggest that systemic metabolic disturbances may drive immune system aging, thereby impairing the body’s overall repair capacity and providing critical insights into the systemic pathological context of diabetic wounds.

At the organizational level, the evidence is more direct. Examinations of clinical samples and diabetic mouse models have consistently confirmed that in the local microenvironment of DFUs, p16, p21, and SASP factors are significantly enriched in skin, adipose, and muscle tissues, clearly linking the accumulation of local senescent cells to impaired healing (10). Molecular network analysis further confirmed that, compared with those in unaffected diabetic foot skin, CDKN1A (p21), CXCL8, and other senescence-related genes are significantly upregulated in DFU, TP53 (p53) is downregulated, and the protein interaction network shows reduced inhibitory interactions and enhanced activity in senescence-related pathways (11). Together, this evidence highlights a clear pathway from systemic metabolic abnormalities to the formation of a local senescent microenvironment, confirming from a systems biology perspective that cellular senescence is a key mediating factor in this disease.

### 2.2 Complexity of senescence: heterogeneity and functional duality

Recent research has revealed that cellular senescence has profound multilevel heterogeneity and a clear dual role in wound healing. At the cellular subpopulation level, heterogeneous senescent cell subpopulations are present during skin wound healing, among which p21^high^ senescent cells (primarily fibroblasts, endothelial cells, and M1 macrophages) release the proinflammatory SASP through the NF-κB pathway, delaying healing and exacerbating fibrosis. Conversely, p16^high^ cells may play a beneficial role (12). At the tissue-specific level, cellular senescence in adipose tissue and pancreatic β-cells in T2DM is tissue specific. Adipose tissue initially accumulates predominantly p21-high cells, and clearance of these cells improves insulin resistance, whereas pancreatic *β*-cells are characterized by enrichment of p16^high^ cells with age and disease progression (13). This discovery challenges generalized senescence intervention approaches and suggests a new strategy for “dual-target tailored senotherapy.”

From the perspective of functional evolution, cellular senescence plays a context-dependent dual role. During physiological repair or embryonic development, acute and controllable cellular senescence promotes tissue repair and homeostasis maintenance through the secretion of SASP factors such as PDGF-AAs. However, under persistent metabolic stress in diabetes, senescence transitions to a chronic and uncontrollable state, leading to abnormal cellular accumulation and excessive secretion of the inflammatory SASP, ultimately resulting in tissue dysfunction and stagnation of healing (14, 15). In summary, cellular senescence in diabetic wounds is a core pathological mechanism driven by metabolic disturbances and is characterized by high heterogeneity and functional duality. This multilayered heterogeneity and functional duality necessitate deeper mechanistic investigations and more precise therapeutic strategies.

## 3 Metabolic disturbances drive cellular senescence

**Figure 1:**
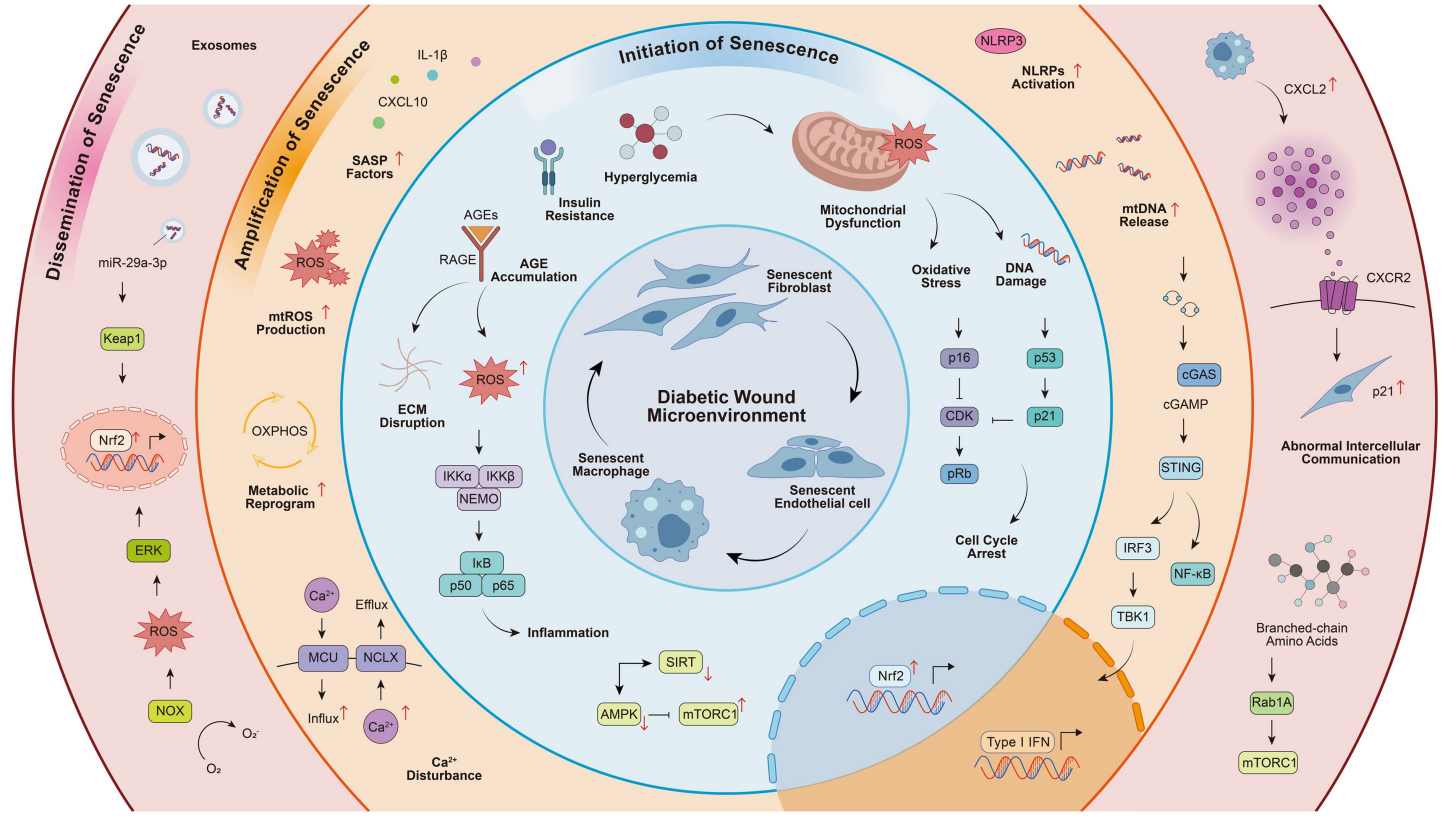
Multilayered mechanisms of metabolism-driven cellular senescence in diabetic wound healing. This figure delineates how systemic metabolic dysregulation drives cellular senescence to impair diabetic wound healing. Centered on a pathological microenvironment with accumulated senescent fibroblasts, endothelial cells, and macrophages, the mechanism unfolds in three concentric layers. The innermost layer illustrates initiation via core metabolic stressors (hyperglycemia, AGEs, insulin resistance), which induce mitochondrial dysfunction, ROS, and DNA damage, activating pathways such as p53/p21. The middle layer shows amplification through dysfunctional mitochondrial networks, where metabolite imbalance, mtROS, and mtDNA release activate immune pathways (e.g., cGAS-STING), creating a vicious cycle. The outermost layer depicts senescence dissemination via the senescence-associated secretory phenotype (SASP), through paracrine signals and exosomes, and links local senescence to systemic aging via circulating factors, collectively stalling tissue regeneration and healing.

In the complex pathological network of chronic diabetic wounds, metabolic disorders constitute the core driving force that initiates and accelerates cellular senescence. The essence lies in the systemic energy metabolism imbalance driven by the combined effects of pancreatic islet β-cell dysfunction (including dedifferentiation and mitochondrial damage) and insulin resistance (16). Notably, the effects of metabolic disorders on cellular function are not limited to energy supply disruption but also involve the direct regulation of gene expression programs through intricate signaling networks. Research has indicated that mTORC1, as the central hub of metabolic sensing, can directly bind to chromatin, phosphorylate transcription factors, and regulate epigenetic modifying enzymes in its nucleus-localized form, deeply participating in cell growth and metabolic processes. Its dysfunction is closely associated with various diseases, including diabetes (17). This series of intracellular events triggered by metabolic dysregulation through complex signal transduction ultimately shapes a difficult-to-reverse senescent microenvironment at the wound site (Figure 1).

### 3.1 Initiation of senescence: direct driving by intracellular metabolic stress

Cellular senescence is initially induced by direct metabolic stress signals within the cell. Mitochondrial dysfunction is a central component of this process, and the resulting energy crisis further leads to the dysregulation of key nutrient-sensing signals such as AMPK/mTOR/SIRT1. These pathways not only are critical for metabolic regulation but also serve as core regulatory hubs of aging. Recent mechanistic studies have shown that in diabetic wounds, high glucose–induced senescent fibroblasts exhibit significantly reduced levels of phosphorylated AMPK. Local application of the AMPK activator A769662 can eliminate senescent cells by inducing autophagy-dependent ferroptosis pathways (18). This finding reveals how abnormal metabolic sensing directly determines the fate of cellular senescence and death.

In addition to energy-sensing dysregulation, the persistent hyperglycemic state itself directly drives cellular senescence through multiple intricate mechanisms. It induces mitochondrial-derived oxidative stress and DNA damage, activating the p53-p21/pRb-p16 signaling pathway and prompting skin fibroblasts, endothelial cells, and other cells to enter a senescent state (19). This process involves complex interactions within signaling networks. For example, the matricellular protein CCN1 can activate the RAC1-NOX1 complex by binding to the cell surface integrins *α*₆*β*₁ and heparan sulfate proteoglycans, generating large amounts of ROS and thereby triggering the DNA damage response and p53 activation. Moreover, through the ROS-dependent ERK/p38 MAPK pathway, the p16^INK4a^/pRb pathway is activated, collectively contributing to fibroblast senescence (20).

In addition, advanced glycation end products (AGEs) are formed and accumulate in large quantities under hyperglycemic conditions, not only directly damaging the structure and function of the extracellular matrix but also activating inflammatory pathways such as NF-κB through binding to their receptor RAGE, exacerbating other aspects of cellular senescence. In DFUs, the vicious cycle between AGEs and ROS has been identified as one of the key pathological mechanisms hindering wound healing (21). Notably, the transcription factor Nrf2 plays a complex role in this process: its sustained activation can induce fibroblast senescence by regulating the matrisome, directly targeting ECM genes such as plasminogen activator inhibitor-1 (PAI-1), and promoting the SASP (22). This discovery reveals a novel role for Nrf2 in the regulation of senescence independent of its traditional antioxidant function.

### 3.2 Amplification of senescence: mitochondrial network dysfunction

In terms of the direct driving mechanisms within cells, senescence signals are further amplified and propagate from the interior of cells to the entire local tissue microenvironment. Mitochondrial network dysfunction plays a critical role as an “amplifier.” Recent studies have revealed that mitochondria form a self-reinforcing vicious cycle with cellular senescence through metabolic reprogramming (e.g., *α*-KG, acetyl-CoA, and NAD^+^ imbalance), mtROS accumulation, mtDNA release-mediated inflammation (cGAS-STING and NLRP3 pathways), and SASP secretion (23). Notably, the expression of MOTS-c, a small peptide encoded by mitochondria, is downregulated during aging and pancreatic β-cell senescence. MOTS-c can reduce the expression of senescence markers (p16, p21, and *γ*-H2AX) and SASP factors (IL-1β and CXCL10) by regulating the mTORC1 signaling pathway, aspartate–glutamate transport, and glutaminolysis-related genes (e.g., *Cd38*, *Grem1*, and *Mdh1b*) while improving mitochondrial oxidative phosphorylation function (24). These findings provide new insights into the communication mechanisms between mitochondria and the nucleus. Additionally, mitochondrial Ca^2+^ signaling dynamically regulates ATP production through MCU complex-mediated matrix Ca^2+^ uptake and NCLX-mediated efflux. Ca^2+^ also participates in mitochondrial quality control by modulating Drp1-mediated mitochondrial fission and Parkin/PINK1-dependent mitophagy (25).

### 3.3 Dissemination of senescence: microenvironment remodeling and systemic propagation

More importantly, the transmission of aging signals extends beyond the realm of cell autonomy. The SASP is the core mechanism through which senescent cells influence the surrounding microenvironment, resulting in the formation of a complex destructive network in diabetic wounds. Studies have shown that delayed healing of diabetic wounds is closely associated with the abnormal accumulation of local senescent cells, where senescent macrophages exhibit polarization abnormalities and secrete SASP-rich CXCR2 ligands (such as CXCL1 and CXCL2). Through paracrine signaling, these SASP factors induce nuclear p21 localization and senescent phenotypes in human dermal fibroblasts (26). These findings reveal that abnormal intercellular communication is a critical mechanism underlying aging-mediated pathological repair in diabetic wounds. Further research highlights the value of CXCR2 as a key therapeutic target for aging-related pathological repair (27), as its antagonists can reverse the SASP-induced transformation of fibroblasts into profibrotic senescent cells.

In terms of intercellular communication, the salivary peptide Histatin 1 (Hst1) exerts its effects through a dual mechanism: first, it regulates NOX-dependent ROS to induce ERK-mediated nuclear translocation of Nrf2, thereby enhancing the antioxidant response; second, it inhibits the excessive formation of mitochondria-associated endoplasmic reticulum membranes (MAMs) mediated by the IP3R1/GRP75/VDAC1 complex, reducing Ca^2+^ transfer from the endoplasmic reticulum to mitochondria and alleviating mitochondrial calcium overload and functional damage (28). Moreover, fibroblast-derived exosomes deliver miR-29a-3p to target the 3’UTR of Keap1 mRNA and promote its degradation, activating the KEAP1/Nrf2 antioxidant pathway (29) and providing a novel mechanism for intercellular communication.

Metabolic dysregulation is a systemic characteristic that drives aging. Research has revealed that branched-chain amino acids (BCAAs) regulate glucose homeostasis through the Rab1A-mTORC1-Pdx1 signaling axis, where Rab1A, as a key amino acid-sensing molecule, activates mTORC1 to increase the stability and nuclear localization of the transcription factor Pdx1 in pancreatic *β*-cells, thereby promoting insulin expression. Clinical data have shown that Rab1A and insulin expression are significantly downregulated in the islets of patients with T2DM and are positively correlated (30). This discovery offers new insights into how metabolic disorders drive cellular dysfunction through nutrient signaling pathways.

Additionally, systems biology research has confirmed that senescent immune cells can induce nonautonomous senescence in distal organ cells through the secretion of SASP components and functional decline (31). This finding elucidates that the difficulty in healing diabetic wounds not only stems from local metabolic microenvironment dysregulation but is also profoundly related to the systemic aging background shaped by systemic metabolic disorders.

### 3.4 Clinical manifestation: DFU/DFI as terminal phenotypes of the “metabolism– senescence axis”

In summary, DFUs and DFIs represent the terminal clinical phenotypes of chronic inflammation and impaired tissue repair in diabetes. The nonhealing nature of these ulcers is essentially a localized manifestation of the dysregulated “metabolism–senescence axis” at the wound site. The pathological microenvironment shaped by hyperglycemia also drives a vicious cycle among microorganisms, host cells, and immune responses. Microbial infection is a key factor leading to chronic wounds. Pathogenic bacteria (such as *Staphylococcus aureus* and *Pseudomonas aeruginosa*) persistently colonize wounds through biofilm formation and virulence factor secretion, directly damaging tissues and severely disrupting the normal transition from the inflammatory phase to the proliferative phase, resulting in a “stagnation” of the repair process (32). Moreover, host metabolic disorders actively exacerbate infection severity. Studies have revealed that in hyperglycemic environments, pathogens such as *Staphylococcus aureus* can adaptively reprogram their metabolism (e.g., enhancing glycolysis) to promote virulence factor expression and biofilm formation, resulting in a transition from a commensal state to an invasive state and exacerbating ulcer chronicity (33). Finally, this process is closely linked to persistent immune dysregulation. In DFU wounds, pattern recognition receptors such as the NLRP3 inflammasome are overactivated, driving macrophages toward M1 phenotype while suppressing their transition to M2 phenotype, leading to unresolved inflammation and impaired tissue regeneration (34).

Each link in this vicious cycle is closely intertwined with the mechanisms described earlier: persistent metabolic stress (high glucose, or AGEs) directly drives cellular senescence; senescent cells further solidify the inflammatory microenvironment and “transmit” the senescent phenotype through SASP-rich CXCR2 ligands; however, the dysregulated immune response is unable to eliminate pathogens whose virulence is enhanced by metabolic reprogramming. Therefore, DFU/DFI can be viewed as a chronic pathological closed loop initiated by metabolic disorders, amplified by cellular senescence, and jointly sustained by immune deficiency and microbial dysbiosis. This integrated perspective clearly suggests that any effective therapeutic strategy must transcend single targets and aim to simultaneously intervene in multiple links along this central axis.

## 4 Targeted senescence therapeutic strategies: from mechanisms to applications

The core mechanism of impaired wound healing in diabetes lies in cell senescence driven by metabolic disturbances, making targeting senescent cells and their related pathways the most promising therapeutic strategy. Current research strategies focus primarily on three major directions: precise clearance of senescent cells, regulation of the harmful SASP, and reprogramming of senescent cell metabolism. These strategies intervene in the senescent microenvironment at different levels, collectively forming a comprehensive therapeutic system targeting the “metabolism–senescence axis” in diabetic wounds.

### 4.1 Senescent cell clearance strategies

Owing to the high heterogeneity of senescent cells, precision clearance has become a core direction in diabetic wound treatment. The evolution of this strategy began with the concept validation of senololytic therapy through the construction of an INK-ATTAC transgenic mouse model, in which p16^INK4A^ was used as a marker to achieve inducible clearance of senescent cells. Research has confirmed that in BubR1 progeria mice, lifelong or late-life clearance of p16^INK4A^-positive senescent cells can delay age-related phenotypes, such as fat loss, sarcopenia, and cataracts (35), providing groundbreaking conceptual validation of the senescent cell clearance strategy.

In the specific pathological environment of diabetic wounds, the concept of precise intervention has been further explored. Research has indicated that not all senescent cells hinder repair; selectively targeting and clearing the p21^high^senescent cell subpopulation (rather than broad clearance) can effectively accelerate wound closure and reduce scar formation in female mice (12). These findings underscore the necessity of precision therapy based on specific biomarkers and cell subtypes, avoiding the potential drawbacks of a “one-size-fits-all” strategy. Further studies propose a more forward-looking “dual-target customized senescence therapy” strategy, aiming to specifically intervene in different senescent cell subpopulations present in various tissues (such as adipose tissue and pancreatic β-cells) in individuals with T2DM (13). This tissue-specific therapeutic approach represents a future direction of senescence treatment.

At the molecular mechanism level, new targets continue to emerge to support precise clearance. Studies have shown that in diabetic foot ulcer tissues and AGE-treated human umbilical vein endothelial cells (HUVECs), the expression of the deubiquitinating enzyme USP7 is upregulated. It stabilizes the p53 protein through deubiquitination, promotes the activation of the p53/p21 pathway, leading to cell cycle arrest and senescence (36). More importantly, the USP7 inhibitor P5091 can activate the p53 pathway, induce the production of ROS in senescent dermal fibroblasts, reduce the mitochondrial membrane potential, decrease the release of SASP factors (IL-1α, IL-1β, etc.), and selectively clear senescent cells (37), providing new candidate targets for the regulation of protein stability by senolytics.

### 4.2 SASP regulation and intercellular communication intervention

Regulating the SASP is crucial for intervening in the aging microenvironment and blocking the transmission of senescence signals. In terms of signaling pathway-targeted therapies, interventions targeting the chemokine receptor CXCR2 have shown significant potential. Research has indicated that senescent macrophages in diabetic wounds exhibit impaired polarization capacity and secrete SASP-rich CXCR2 ligands (such as CXCL1/2), which induce senescence and fibrosis in dermal fibroblasts through paracrine mechanisms, thereby hindering repair (26). The application of the CXCR2 antagonist SB265610 can effectively reverse this pathological process, reducing macrophage senescence and neutrophil infiltration and thereby accelerating the healing of diabetic mouse and human ex vivo wounds (27). This study revealed that CXCR2 is a key target that mediates senescence-associated pathological repair in diabetic wounds.

In terms of cell fate and microenvironment regulation, the melanocortin 1 receptor (MC1R) has been identified as a key molecule that affects the healing outcomes of acute and chronic skin wounds. Research clearly demonstrates that dysregulation of the POMC–MC1R axis is a common feature of chronic wounds such as DFUs and pressure ulcers. Loss of MC1R function leads to delayed wound re-epithelialization and increased numbers of neutrophil extracellular traps (NETs). By constructing an aged and oxidative stress-induced chronic wound mouse model, topical application of the MC1R agonist BMS-470539 was confirmed to reduce exudation and NET formation and promote angiogenesis and re-epithelialization, whereas in acute wounds, it can increase collagen deposition (by lowering the type I/III collagen ratio) and reduce scar formation (38). This discovery reveals the core regulatory role of MC1R in wound repair and provides a new direction for regulating the microenvironment through receptor-targeted approaches.

### 4.3 Metabolic reprogramming

Targeting the metabolism of senescent cells is a strategy for improving their function at the root level. Pyruvate dehydrogenase kinase 4 (PDK4) plays a key role in this process. PDK4 reduces ROS production through metabolic reprogramming (enhancing glycolysis and inhibiting mitochondrial respiration), thereby activating the Yes-associated protein (YAP) pathway (promoting nuclear translocation) and inhibiting c-Jun N-terminal kinase (JNK) phosphorylation, decreasing the expression of senescence markers such as p53 and p21, as well as SASP factor expression, and improving the senescent phenotype of fibroblasts (39). Further research revealed that PDK4 can increase succinate levels and inhibit PHD2 enzyme activity to stabilize the HIF-1α protein while increasing HIF-1α mRNA expression and promoting fibroblast proliferation, migration, and myofibroblast differentiation (40). These findings reveal a sophisticated regulatory network between metabolism and epigenetics, providing a theoretical basis for reversing cellular senescence phenotypes through metabolic interventions.

### 4.4 Ferroptosis signaling

Ferroptosis is an iron dependent, lipid peroxidation-driven form of programmed cell death, and its role in the pathogenesis of diabetic wounds has garnered increasing attention. Systematic reviews suggest that regulating ferroptosis is a potential strategy for improving T2DM and its complications, clarifying the central role of ferroptosis in *β*-cell dysfunction, insulin resistance, and various complications (41). In diabetic wounds, high glucose–induced senescent fibroblasts exhibit resistance to ferroptosis. The key mechanism lies in the downregulation of NCOA4, a critical molecule in ferritinophagy, leading to impaired ferritin degradation and insufficient intracellular free iron levels (42). NCOA4 overexpression can restore the ferritin degradation pathway and increase free iron levels, thereby resensitizing senescent fibroblasts to ferroptosis and offering a novel approach to eliminate these stubborn cells. This discovery successfully establishes an intrinsic link between “iron metabolism–ferroptosis–senescent cell clearance.” Notably, topical application of the AMPK activator A769662 can clear senescent cells by inducing NCOA4-mediated ferritinophagy. The specific mechanism involves AMPK activation to upregulate NCOA4 expression, subsequently promoting ferritinophagy and enhancing ferritin degradation and free iron release, ultimately triggering ferroptosis (18). This finding echoes the previously mentioned role of AMPK in energy sensing, collectively forming a complex regulatory network from the sensing of metabolic stress to the execution of cell clearance.

### 4.5 Targeting the wound microbiome

The aforementioned strategies focus on correcting the host’s intrinsic “metabolism-senescence” program. However, the refractory nature of DFUs stems from a more complex and dysregulated ecosystem, in which disruption of the wound microbiome is a key external factor that drives and maintains the chronic aging microenvironment. Dysbiosis not only directly triggers infections but also indirectly exacerbates local cellular senescence and dysfunction through persistent inflammation, impaired re-epithelialization, and other mechanisms. Therefore, targeting the microbiome aims to add to the critical external driver, providing a novel intervention dimension to break the chronicity cycle and shifting the treatment paradigm from “broad-spectrum sterilization” to “ecological remodeling.”

Precision intervention begins with an in-depth understanding of DFU microbial characteristics. Metagenomic analyses revealed that the DFU microbiome has two key features: widespread biofilm formation and an imbalanced microbial community structure. Its composition dynamically correlates with clinical severity: as infection worsens (increasing PEDIS grade), microbial diversity decreases, and pathogenic communities (e.g., Gammaproteobacteria) become enriched, whereas potentially beneficial commensals (e.g., *Corynebacterium*) diminish (43, 44). These findings provide a basis for precise stratification and intervention on the basis of microbial features.

Although widely used, traditional local antibacterial strategies (such as silver- or iodine-containing dressings) have limited high-level evidence for promoting healing and carry risks of drug resistance (45). Therefore, new strategies aimed at regulating rather than eradicating the microbiota have emerged as cutting-edge approaches. For example, a smart hydrogel loaded with selenium nanoparticles can generate antioxidant selenoproteins *in situ* at the wound site, selectively inhibiting the activity of pathogenic bacteria such as *Staphylococcus aureus* while promoting the colonization of beneficial bacteria such as *Lactobacillus*. It also synergistically induces macrophage polarization toward the reparative M2 phenotype, achieving synergistic effects on “antibacterial–probiotic–immunomodulatory” functions (46).

In summary, the aforementioned strategies, ranging from precise elimination, secretion regulation, and metabolic reversal to the utilization of novel death mechanisms, form a multilayered, multitarget intervention network. Moreover, therapeutic strategies targeting the wound microbiome add an indispensable external dimension to this intervention system. It does not replace host-directed strategies but rather synergizes and complements them, which are jointly committed to reshaping a microenvironment conducive to healing. All these strategies not only directly stem from an in-depth understanding of the “metabolism–senescence axis” mechanism but also provide a clear action framework and integration opportunities for natural products to leverage their multicomponent, multipathway synergistic therapeutic advantages.

## 5 Multitarget intervention strategies for natural products and traditional formulations

**Figure 2:**
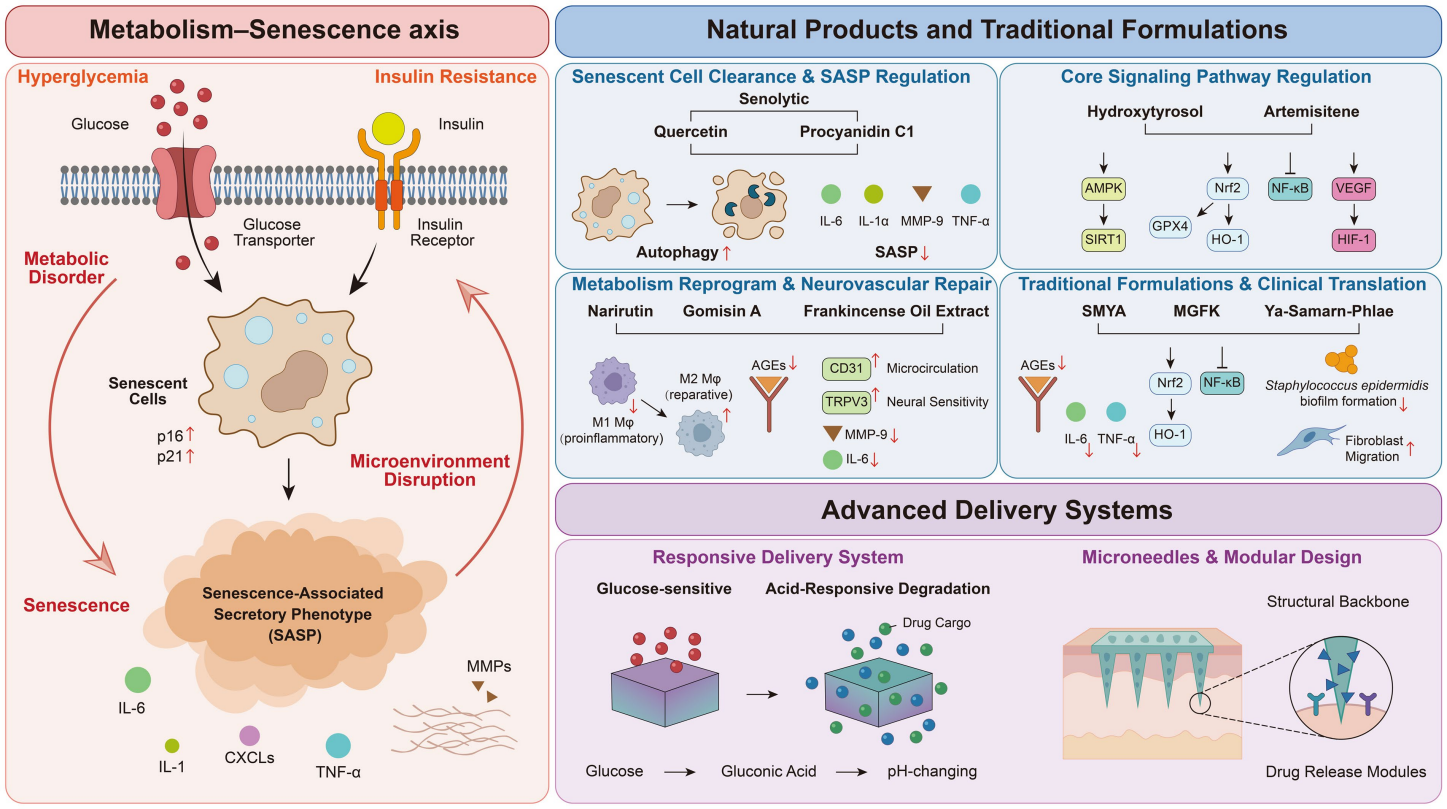
Targeting the metabolism-senescence axis in diabetic wounds with natural product-based multilevel interventions. This figure illustrates the integrated “metabolism-senescence” perspective on the healing of chronic diabetic wounds. The left side shows the core pathological axis in which metabolic disorders (such as hyperglycemia and insulin resistance) drive cellular senescence and disrupt the microenvironment through the SASP, resulting in a vicious cycle. The right side systematically demonstrates the multilevel intervention strategies of natural products, including direct clearance of senescent cells, synergistic regulation of core signaling pathways, immune–metabolic reprogramming, and systemic integration of traditional compound formulations. These strategies are empowered through intelligent delivery systems (such as responsive hydrogels and microneedles) to achieve precise intervention, ultimately synergistically promoting wound regeneration and repair.

Owing to their multicomponent, multitarget, and multipathway characteristics, natural products have immense potential for synergistic intervention in complex network systems. Compared with single-target synthetic drugs, they possess unique advantages in regulating the “metabolism–senescence axis.” Systematic studies have confirmed that active ingredients in traditional Chinese medicine, such as *Scutellaria baicalensis*, curcumin, and *Panax notoginseng*, can synergistically regulate at least seven core signaling pathways, including the Nrf2/ARE, NF-κB, and PI3K/Akt pathways, thereby exerting synergistic therapeutic effects across multiple pathological aspects, such as antioxidant, anti-inflammatory, proangiogenic, and extracellular matrix remodeling (47). This synergistic mechanism of multiple pathways provides a solid theoretical foundation for natural products to directly intervene in metabolic disorders and aging processes, systematically regulate the wound microenvironment, and establish a comprehensive intervention strategy system ranging from clearing senescent cells to remodeling the repair microenvironment (Figure 2; Table 1).

**Table 1:** Multitarget intervention mechanisms of natural products and traditional formulations in diabetic wound healing.

Intervention strategy and molecular focus	Representative agent (Source)	Detailed molecular targets and interaction	Elaborated mechanisms of action and experimental evidence
Direct Senolytic Intervention and SASP suppression	Quercetin (Senolytic cocktail component)	• Primary target: Senescent cells (e.g., in dermis and adipose). • SASP factors: IL-1α, IL-6, MMP-9.	• Senolysis: Effectively reduces senescent cell burden in human diabetic adipose and skin tissues. • SASP attenuation: Significantly lowers circulating levels of key pro-inflammatory and tissue-destructive SASP factors in patient plasma.
	Resveratrol, Berberine (AMPK activators)	• Core Pathway: AMPK/SIRT1 signaling axis. • Cellular Process: Autophagy machinery.	• Metabolic sensing: Activates AMPK, improving cellular energy status under high-glucose stress. • Pro-survival autophagy: Upregulates autophagy, clearing damaged organelles and delaying the initiation of cellular senescence.
	Procyanidin C1 (PCC1) (Novel senolytic)	• Specific Target: P16^INK4a^ senescent cells. • Key pathway: NF-κB signaling.	• Selective clearance: Induces apoptosis specifically in senescent cells, showing potential for targeting senescence subpopulations accumulated under metabolic disorder. • SASP Inhibition: Suppresses NF-κB transactivation, downregulating TNF-*α* and IL-6 secretion. • Functional recovery: Restores proliferative capacity of dermal fibroblasts and angiogenic function of endothelial cells. Demonstrated superior efficacy and safety over ABT-263 in both type I and II diabetic mouse Models.
Synergistic regulation of core metabolism and senescence signaling pathways	Hydroxytyrosol (Hy) (Olive derivative)	• Multitarget engagement: Nrf2, NF-κB p65 subunit, Akt. • Downstream effectors: HO-1, VEGF-A, HIF-1α.	• Antioxidant: Activates the Nrf2/HO-1 pathway, enhancing cellular resistance to metabolic oxidative stress. • Anti-inflammatory: Inhibits NF-κB nuclear translocation, reducing pro-inflammatory cytokine production. • Angiogenic: Promotes Akt phosphorylation and upregulates VEGF-A/HIF-1α expression, stimulating new blood vessel formation. A prime example of “antioxidant-anti-inflammatory-pro-angiogenic” synergy.
	Artemisitene (ATT) (Ferroptosis inhibitor)	• Central pathway: Nrf2/GPX4 axis. • Ferroptosis marker: ACSL4.	• Ferroptosis suppression: Binds with high affinity to Nrf2 and GPX4, activating Nrf2 signaling and upregulating the key antioxidant enzyme GPX4. • Lipid peroxidation control: Downregulates pro-ferroptotic protein ACSL4. • Cytoprotection: Reduces mitochondrial ROS generation and rescues mitochondrial membrane potential in HUVECs under high glucose, effectively blocking ferroptosis.
Immunometabolic reprogramming and neurovascular repair	Narirutin (Citrus flavonoid)	• Metabolic SWITCH: AMPK/Mfn2 pathway. • Immunophenotype: Macrophage polarization.	• Metabolic reprogramming: Activates AMPK, promoting Mfn2-dependent mitochondrial fusion and shifting macrophage metabolism from glycolysis (M1-state) to oxidative phosphorylation (M2-state). • Pro-repair Polarization: Drives macrophages toward the healing M2 phenotype, facilitating tissue repair.
	Gomisin A (Schisandra extract)	• Inflammatory Axis: TLR4-p38 MAPK-IL6. • Metabolic Sensor: Akt. • Precursor: AGEs.	• Anti-glycation: Reduces the formation of AGEs. • Anti-inflammatory: Suppresses the TLR4-p38 MAPK-IL6 signaling cascade. • Insulin Sensitization: Enhances Akt phosphorylation, improving insulin signaling. • Pro-healing: Collectively promotes collagen deposition and angiogenesis.
	Frankincense extract (Boswellia)	• Vascular marker: CD31 (PECAM-1). • Ion Channel: TRPV3. • Inflammatory Mediators: β-catenin, MMP-9, COX-2.	• Vascular repair: Upregulates CD31, improving microcirculation and endothelial function. • Neural recovery: Increases TRPV3 expression, restoring peripheral neural sensitivity. • Anti-inflammatory: Downregulates β-catenin signaling, MMP-9 (extracellular matrix degradation), and COX-2 (prostaglandin synthesis), mitigating excessive inflammation. Comprehensively aids in restoring foot sensation and motor function.
Systemic therapy with traditional formulations	Si-Miao-Yong-An decoction (SMYA) (TCM Formula)	• Receptor pathway: AGE-RAGE. • Cytokine targets: TNF, IL-6.	• Network Pharmacology: Core components (Quercetin, Kaempferol) bind with high affinity to TNF and IL-6. • Mechanism: Inhibits AGE-RAGE signaling, leading to significant downregulation of *Tnf* and *Il1b* gene expression, thereby suppressing the diabetic inflammatory cascade and promoting healing.
	Mai-Guan-Fu-Kang tablets (MGFK) (TCM formula)	• Multipathway Core: Nrf2/HO-1, NF-κB, VEGF/NO.	• Systemic regulation: Synchronously activates the Nrf2 antioxidant pathway, inhibits the NF-κB inflammatory pathway, and elevates VEGF/NO levels to promote angiogenesis. • Functional Outcome: Comprehensively improves lower limb blood supply and sciatic nerve function in diabetic Models.
	Hua-Fu-Zai-Sheng method (e.g., Shengji Ointment + Bromelain) (Integrated TCM protocol)	• Clinical Focus: Severe DFU (tendon-exposed). • Microecological target: Wound microbiota. • Metabolic pathway: Phenylpropanoid biosynthesis.	• Clinical efficacy (RCT): Significantly increases granulation coverage, shortens healing time, and shows good safety for severe DFU. • Microbiome remodeling: Integrated omics reveals it reduces pathogenic bacteria (e.g., *Erysipelatoclostridium*) and increases beneficial bacteria (e.g., *Cetobacterium*), reshaping the wound ecological niche. • Metabolic reprogramming: Regulates key metabolic pathways (e.g., phenylpropanoid biosynthesis) to improve the wound metabolic microenvironment, exemplifying the modern connotation of “strengthening vital qi and eliminating pathogenic factors” by modulating host–microbe interactions and local homeostasis.
	Ya-Samarn-Phlae (Thai Traditional Formula)	• Multicomponent Action: Bacterial biofilms, free radicals, fibroblast motility.	• Anti-biofilm: Inhibits *Staphylococcus epidermidis* biofilm formation. • Antioxidant: Scavenges nitric oxide free radicals. • Cell migration: Enhances fibroblast migration. • Clinical Evidence: As an adjuvant to standard care, achieved a 76% complete ulcer healing rate over 12 weeks, vastly outperforming the control group (16%).
	Integrated chinese-western protocol (e.g., Yanghe decoction + Sijunzi decoction)	• Systemic modulation: Multiple inflammatory and repair pathways. • Clinical focus: Critical limb ischemia and severe infection in comorbid conditions.	• Case Study: Achieved complete wound healing, limb salvage, and dramatic reduction in systemic inflammation (87% drop in CRP) in an outpatient setting for a high-risk patient with uremic diabetic foot gangrene. • Value: Demonstrates a cost-effective paradigm for complex, refractory wounds.

↑Indicates upregulation/activation; ↓Indicates downregulation/inhibition. This table details the multitarget and synergistic mechanisms by which natural products and formulations intervene in key pathological processes of diabetic wounds—including cellular senescence, oxidative stress, inflammation, impaired angiogenesis, and immune dysfunction—provides a robust scientific rationale for their therapeutic application.

ACSL4, Acyl-CoA synthetase long chain family member 4; AGEs, advanced glycation end products; Akt, protein kinase B; AMPK, AMP-activated protein kinase; COX-2, cyclooxygenase-2; CRP, C-reactive protein; HIF-1α, hypoxia-inducible factor 1-alpha; HO-1, heme oxygenase 1; HUVECs, human umbilical vein endothelial cells; IL, interleukin; MAPK, mitogen-activated protein kinase; Mfn2, mitofusin-2; MMP-9, matrix metalloproteinase-9; NF-κB, nuclear factor Kappa-Light-Chain-Enhancer of activated B cells; NO, nitric oxide; Nrf2, nuclear factor erythroid 2–related factor 2; PECAM-1, platelet endothelial cell adhesion molecule-1; ROS, Reactive ox activated B cells.

### 5.1 Targeting senescent cell clearance and SASP regulation

Directly and precisely clearing senescent cells or inhibiting their harmful secretory phenotype is the most straightforward strategy for natural products to intervene in the “metabolism–senescence axis.” As a component of the classic senolytic cocktail (D + Q), quercetin has been shown to effectively reduce the senescent cell burden in the adipose tissue and skin of diabetic patients and lower the plasma levels of SASP factors such as IL-1α, IL-6, and MMP-9 (48); these findings have been validated from the laboratory to the clinic. Additionally, natural components such as resveratrol and berberine can activate the AMPK/SIRT1 pathway, fundamentally improving energy metabolism disorders and promoting autophagy, thereby delaying the initiation of cellular senescence (49).

A significant advancement is the discovery of the novel natural-source senolytic agent procyanidin C1 (PCC1). PCC1 selectively clears P16^+^ senescent cells by inhibiting the NF-κB signaling pathway to downregulate SASP factors such as TNF-*α* and IL-6, effectively restoring fibroblast and endothelial cell functions. Compared with the traditional synthetic senolytic drug ABT-263, PCC1 accelerated wound healing and demonstrated greater safety and therapeutic efficacy in type II diabetic mouse models (50). These studies indicate that natural products have the potential to precisely target and eliminate specific senescent cell subpopulations that accumulate under metabolic disorder conditions.

### 5.2 Synergistic regulation of Core metabolism and the aging signaling pathway

The core advantage of natural products lies in their ability to synergistically intervene in multiple upstream and downstream signaling pathways that drive aging. As an active component in olive oil, hydroxytyrosol (Hy) exerts multidimensional therapeutic effects by binding with high affinity to core targets such as Nrf2 and NF-κB. On the one hand, it activates the Nrf2/HO-1 pathway to increase the cellular antioxidant capacity against metabolic oxidative stress. On the other hand, it suppresses NF-κB-mediated inflammatory responses while simultaneously increasing Akt phosphorylation and vascular endothelial growth factor A (VEGF-A)/HIF-1α expression to promote angiogenesis (51). This synergistic “antioxidant-anti-inflammatory-angiogenic” mechanism precisely intervenes in multiple downstream stages of metabolic damage.

In the regulation of ferroptosis, the cell death mechanism is closely associated with metabolic disorders, and natural products also have unique value. They primarily activate the Nrf2 pathway to upregulate key targets such as GPX4 and HMOX1 while modulating pathways such as AdipoR1/AMPK and SIRT1/HMGB1 to suppress ferroptosis-related damage (41). For example, the artemisitene (ATT) derived from the herb *Artemisia annua* binds with high affinity to Nrf2 and GPX4, effectively reducing ROS generation, restoring mitochondrial function, inhibiting ACSL4 expression, and enhancing GPX4 activity, thereby blocking the ferroptosis process under high-glucose conditions (52).

### 5.3 Metabolism reprogramming and neurovascular repair

Natural products can also improve the aging-related microenvironment by reprogramming the metabolism of immune cells. Citrus peel-derived narirutin activates AMPK/Mfn2 signaling, regulating macrophage metabolism and shifting from glycolysis to oxidative phosphorylation, thereby promoting macrophage polarization from the proinflammatory M1 type to the reparative M2 type and ultimately accelerating wound healing (53). The active component Gomisin A from *Schisandra chinensis* (Turcz.) Baill., on the other hand, reduces the generation of advanced glycation end products, inhibits the TLR4/p38 MAPK/IL-6 inflammatory pathway, and upregulates Akt phosphorylation to improve insulin sensitivity, promoting repair across multiple dimensions (54).

In neurovascular and vascular repair, frankincense oil extract upregulated CD31 expression to improve microcirculation and repair vascular endothelial damage while increasing TRPV3 expression to restore neural sensitivity. It also downregulates β-catenin, MMP-9, and COX-2 to suppress excessive inflammation, thereby restoring sensory and motor functions comprehensively (55). These findings indicate that natural products can be used to systematically treat coexisting vascular and neuropathic disorders in diabetic wounds.

### 5.4 Systematic integration of traditional formulations and clinical translation

Traditional Chinese medicine (TCM) compound formulas, which are based on the compatibility theory of “sovereign, minister, assistant, and envoy,” demonstrate stronger systemic regulatory advantages in the treatment of diabetic wounds. Modern research has gradually revealed their scientific connotations through technologies such as network pharmacology, molecular docking, and molecular dynamics simulation. The core active components of Simiao Yong’an Decoction (SMYA), quercetin and kaempferol, can bind with high affinity to inflammatory targets such as TNF and IL-6. By inhibiting the AGE-RAGE signaling pathway, they effectively downregulate the expression of key inflammatory factors such as Tnf and Il1b, thereby promoting the healing of diabetic wounds (56). Maiguan Fukang tablets (MGFKs), which contain components such as *Salvia miltiorrhiza* and frankincense, can simultaneously activate the Nrf2/HO-1 pathway for antioxidation, inhibit NF-κB-mediated inflammation, and increase VEGF/NO levels to improve the lower limb blood supply and sciatic nerve function (57). Systematic reviews have confirmed that TCMs and their compound formulas, such as those of Astragalus and Angelica sinensis, can precisely regulate immune cells such as T cells and macrophages, intervene in key pathways such as the PI3K/Akt and NF-κB pathways, and achieve bidirectional anti-inflammatory and promote-repair effects. Moreover, the clinical efficacy of integrated Chinese and Western medicine protocols is significantly superior to that of single therapies (58).

In terms of clinical efficacy, high-quality studies have provided conclusive evidence. For example, a multicenter randomized controlled trial demonstrated that the “Hua Fu Sheng Ji” method, which is based on the combination of the traditional Chinese medicine Shengji ointment and bromelain, significantly increased granulation tissue coverage in tendon-exposed DFUs, shortened healing time, and exhibited good safety, providing reliable TCM treatment for severe DFU (59). Another systematic review confirmed that TCM exerts proangiogenic, antioxidant, anti-inflammatory, and antibacterial effects through multitarget regulation of key pathways and that topical formulations have more substantial clinical validation than oral preparations do (60). This advantage is particularly prominent in complex cases: for extremely high-risk and complex cases of uremia combined with diabetic foot gangrene, the integrated Chinese–Western medical approach (e.g., Yanghe Decoction combined with Sijunzi Decoction, staged debridement, and local analgesia) successfully achieved complete wound healing, significant improvement in inflammatory markers (87% reduction in CRP), and limb preservation in outpatient settings, with treatment costs far lower than those of purely Western inpatient or amputation approaches (61). This successful case highlights an extremely valuable real-world paradigm for the application of natural products and compounds in complex clinical scenarios.

In addition to traditional mechanisms and macroscopic therapeutic effects, cutting-edge research is needed to elucidate the “holistic regulation” of TCMs from the perspectives of microecology and metabolomics. Integrated omics analysis of “Huafu Zaisheng” method (62) revealed that its remarkable wound-healing effects stem not only from direct tissue regeneration but also from its close association with the local microbiota ecology of wounds—significantly reducing the abundance of pathogenic bacteria such as Erysipelatoclostridium while increasing the abundance of beneficial bacteria such as Cetobacterium. Moreover, this therapy regulates key metabolic pathways, such as phenylpropanoid biosynthesis, improving the metabolic microenvironment of the wound. This profoundly reveals the modern connotation of TCM’s “strengthening vital qi and eliminating pathogenic factors” philosophy: by modulating host–microbe interactions and local metabolic homeostasis, it fundamentally breaks the vicious cycle of chronic infection and tissue nonhealing.

Traditional international medicine has also demonstrated immense potential. The traditional Thai compound Ya-Samarn-Phlae contains various active ingredients, such as arecoline, curcumin, and *α*-mangostin flavonoids, which synergistically inhibit the formation of *Staphylococcus epidermidis* biofilms, scavenge nitric oxide free radicals, and promote fibroblast migration. As an adjunct to standard wound care, it achieved a complete diabetic ulcer healing rate of 76% within 12 weeks, which was significantly superior to that of the control group (16%) (63).

## 6 Innovative delivery systems empower natural product therapies

Although natural products have multiple advantages in terms of their ability to intervene in the “metabolism–senescence axis,” their inherent physicochemical property deficiencies, such as poor water solubility, low chemical stability, insufficient systemic bioavailability, and lack of targeting, remain critical bottlenecks limiting their translation to clinical efficacy. The rise of innovative delivery systems has provided revolutionary solutions to address these challenges. By increasing stability, increasing local concentration, achieving spatiotemporally controlled release, and generating synergistic biological effects with natural products, delivery systems can significantly increase the therapeutic potential of natural products (39). More importantly, these systems can be designed to respond precisely to the unique “metabolic disorder microenvironment” of diabetic wounds (e.g., hyperglycemia, acidosis, and oxidative stress), thereby transforming pathological features into opportunities for precision therapy and maximizing the multitarget characteristics of natural products at the lesion site (Table 2).

**Table 2:** Advanced delivery systems for enhancing natural product (NP) therapeutics in diabetic wounds.

System category and strategy	Specific formulation (composition and np)	Technical design and trigger mechanism	Key functional outcomes and experimental evidence
Stimuli-responsive “smart” systems	ROS-scavenging nanocomposite hydrogel: GelMC/PVA-UIO-66-NH₂@Quercetin	• Mechanism: ROS-cleavable boronic ester bonds. • Characteristic: Sustained, on-demand Que. release under high ROS.	• Antioxidant: Scavenged multiple ROS species. • Immunomodulation: Promoted M2 macrophage polarization (↓ IL-6/TNF-α, ↑ IL-10). • Angiogenesis: Enhanced HUVEC migration and tube formation (↑ VEGFA expression).
	Cascade Reaction-driven hydrogel: sprayable GelMA with ZIF-8@CeO₂ and GOx	• Mechanism: Enzyme (GOx)-initiated acidification → pH-triggered ZIF-8 degradation. • Feature: Self-amplifying, closed-loop therapeutic cycle.	• Metabolic Regulation: Consumed excess glucose. • Antibacterial: Released Zn^2+^. • Antioxidant/Anti-AGE: CeO₂ nanozyme activity (↓ ROS, ↓ AGEs).
	NIR-triggered synergistic system: CIZ@G Thermosensitive Hydrogel (Chlorogenic Acid)	• Mechanism: NIR laser (808 nm) → photothermal effect → gelation and drug release. • Composition: Self-assembled CA/ICG/Zn^2+^ nanoparticles.	• Antibacterial: Potent photothermal/photodynamic therapy + Zn^2+^/CA synergy. • Antioxidant: CA scavenges residual ROS. • Pro-healing: Zn^2+^ upregulated VEGF/CD31.
Microneedles (MNs) and modular design	Hydrogel MN: LSI-GCA MN with Isoliquiritigenin (ISO)	• Mechanism: pH-responsive ISO release triggered by the alkaline wound environment; visual pH monitoring via anthocyanin. • Design: Core-shell structure for diagnosis and therapy.	• Antioxidant: Activated NRF2 pathway. • Anti-inflammatory: Suppressed NF-κB pathway (↓ TNF-α, IL-6). • *In Vivo* Efficacy: Accelerated wound closure in diabetic rats.
	Soluble Polyvinylpyrrolidone MN Patch (loaded with *Sanguis draconis* and *Salvia miltiorrhiza* extracts)	• Mechanism: Dissolves upon insertion into the skin, releasing traditional Chinese medicine extracts. • Advantage: Minimally invasive, bypasses the stratum corneum, achieves high local drug concentration.	• Pro-healing: Improves the hyperglycemic microenvironment, promotes the migration of keratinocytes and fibroblasts. • Enhanced Microcirculation: Increases wound blood flow velocity. • Efficacy: Synergistically accelerates diabetic wound healing.
	Carrier-free Coassembled NPs in Gel: Nar-Cur NPs (NC) in Thermosensitive Gel (NC@Gel)	• Mechanism: π-π stacking and H-bonding self-assembly; no synthetic carrier. • Advantage: High drug loading, simplified preparation, improved biocompatibility.	• Mitochondrial Protection: Restored membrane potential, alleviated Ca^2+^ overload. • Synergistic Signaling: Coactivated Nrf2/HO-1 (↑) and inhibited NF-κB (↓).
	Multimodular Hydrogel: CS&Gel-PA&Fe-QT&PElip Hydrogel	• Design: Discrete functional modules: structural base, QT-liposomes (drug), PA-Fe complex (auxiliary function). • Crosslinking: Dynamic Schiff base and H-bonds.	• Multifunctional: Combined antioxidant (QT), antibacterial (PA-Fe, CS), and anti-inflammatory actions. • Self-adaptive: Excellent tissue adhesion and self-healing properties.
Advanced Hydrogel Platforms	Dual pH/ROS-responsive hydrogel: Polysaccharide hydrogel with Curcumin/Tempol liposomes	• Mechanism: Dual-responsive degradation for programmed drug release. • Payload: Combination of two antioxidant NPs.	• Comprehensive healing: Effective ROS scavenging, mitochondrial protection, M2 polarization, antibacterial activity, and hemostasis. • *In vivo* validation: Enhanced epithelial regeneration, collagen deposition, and angiogenesis in diabetic rat models.
	Bioactive material-based platform: Silk Fibroin Dressing (e.g., loaded with Curcumin)	• Strategy: Uses innate bioactivity of natural polymer (SF) as a functional carrier. • Versatility: Adaptable to various formats (films, hydrogels).	• Antibacterial: Membrane disruption mechanism. • Pro-healing: Modulated NF-κB/PI3K pathways; promoted fibroblast proliferation and angiogenesis with its natural structure potentially contributing to signaling modulation.
	Next-generation Vesicle-Hydrogel hybrids: hydrogel loaded with Viola Yedoensis Makino-Derived Exosome-like Nanovesicles	• Platform: Plant-derived nanovesicles as natural NP carriers within a bioadhesive hydrogel.	• Immunomodulation: Promoted M2 polarization via NF-κB inhibition. • Specialized Healing: Promoted tissue repair by enhancing osteogenesis.
	Exosome-Enhanced Hydrogel: Thermosensitive Gel with MSCs-exosomes loaded with paeonol	• Platform: Combines synthetic NP (paeonol), biological vesicles (exosomes), and smart material (gel). • Molecular mechanism: Exosome-mediated miRNA (miR-424-5p) delivery.	• Cellular reprogramming: Promoted fibroblast EMT and angiogenesis. • Efficacy: Significant acceleration of wound closure in diabetic mice.

↑Indicates upregulation/activation; ↓Indicates downregulation/inhibition. These advanced systems address NP limitations (e.g., poor solubility, low bioavailability) through sophisticated engineering strategies, enabling targeted, efficient, and synergistic multimodal therapy for diabetic wounds.

CA, chlorogenic acid; Cur, curcumin; EMT, epithelial–mesenchymal transition; GOx, glucose oxidase; HUVEC, human umbilical vein endothelial cell; ICG, indocyanine green; IL, interleukin; MNs, microneedles; MSCs, mesenchymal stem cells; Nar, naringenin; NF-κB, nuclear factor Kappa-Light-Chain-Enhancer of activated B cells; NIR, near-infrared; Nrf2, nuclear factor erythroid 2–related factor 2; NPs, natural products; Que., quercetin; ROS, reactive oxygen species; TNF-*α*, tumor necrosis factor-alpha; VEGF(A), vascular endothelial growth factor (A).

### 6.1 Intelligent responsive delivery systems

The core breakthrough of intelligent responsive delivery systems lies in their ability to convert the adverse microenvironment of diabetic wounds (e.g., hyperglycemia, acidic pH, and excessive reactive oxygen species production) into “switches” that trigger precise drug release (64), thereby enabling on-demand delivery of natural products.

ROS-responsive systems are the primary representatives and directly target the core pathological feature of oxidative stress in wounds. For example, a nanocomposite hydrogel dressing loaded with quercetin (Que) (GelMC/PVA-UIO-66-NH₂@Que), cross-linked via dynamic borate ester bonds, can undergo bond cleavage in high-ROS environments, enabling the precise release of Que. The released Que. not only effectively scavenges ROS but also regulates macrophage polarization toward the reparative M2 phenotype (manifested by downregulation of IL-6, TNF-*α* and upregulation of IL-10) and promotes the HUVECs migration and the VEGF-A expression (65). This processing directly transforms the metabolic injury marker “excessive ROS” into a signal that initiates the “antioxidant–anti-inflammatory–pro-repair” program.

A more sophisticated strategy is reflected in the metabolic cascade response system. The sprayable GelMA hydrogel (zcgG) constructed by cascade nanozymes (ZIF-8@CeO₂) and glucose oxidase (GOx) establishes an intrinsic “sensing-feedback” therapeutic loop: GOx catalyzes the conversion of excess glucose in the wound into gluconic acid, leading to a localized decrease in pH, which triggers the acid-responsive degradation of ZIF-8, thereby promoting the synchronous release of antibacterial Zn^2+^ and CeO₂ nanozymes that combine ROS scavenging with the inhibition of AGEs (66). This “glucose consumption–antibacterial–antioxidant–antiglycation” cascade strategy precisely targets the core metabolic issue of diabetic wounds, where hyperglycemia and glycation damage coexist.

In addition, physical signal response systems (such as light, heat, and magnetism) provide an externally controllable and precise manipulation dimension for natural product therapy, compensating for the limitations of relying solely on biological microenvironment signals. For example, the chitosan-based thermosensitive hydrogel CIZ@G, loaded with CIZ nanoparticles self-assembled from chlorogenic acid (CA), indocyanine green (ICG), and zinc ions (Zn^2+^) under 808 nm near-infrared laser irradiation, can simultaneously achieve photothermal/photodynamic antibacterial effects and accelerate gel formation. During this process, the natural product CA can scavenge ROS, synergizing with Zn^2+^ to inhibit *Staphylococcus aureus* and *Escherichia coli*; moreover, Zn^2+^ can also upregulate VEGF/CD31 expression to promote angiogenesis (67). This combination of photothermal response and natural phenolic acid molecules achieves multiple synergies between external physical triggers and internal biological effects.

### 6.2 Microneedles and modular design

To overcome the skin barrier and achieve higher local drug concentrations, microneedle (MN) technology provides a minimally invasive and highly efficient solution (68). Its core enabling capability lies in its ability to create micron-sized channels that deliver active ingredients directly to the dermis. pH-responsive hydrogel microneedles (LSI-GCA) loaded with isoliquiritigenin (ISO) form an intelligent system that integrates “diagnosis” and “treatment.” It utilizes *Lycium barbarum* polysaccharide stearate micelles to load ISO, with chitosan/anthocyanin as the backing layer for real-time pH monitoring. When an alkaline wound environment is sensed, the MNs intelligently release ISO, enhancing antioxidant defenses by activating the Nrf2 pathway and inhibiting the NF-κB pathway to downregulate TNF-*α* and IL-6 expression, thereby exerting therapeutic effects (69). Moreover, a soluble polyvinylpyrrolidone microneedle patch loaded with Sanguis draconis and *Salvia miltiorrhiza* extract can improve the high-glucose environment, promote keratinocyte and fibroblast migration, increase the blood flow velocity and microcirculation of the wound, and synergistically accelerate diabetic wound healing, demonstrating the advantages of microneedle systems in the delivery of traditional Chinese herbal compound formulations (70).

Modular design enables flexible functional combinations and high customization at both the molecular and material levels. At the molecular level, naringenin (Nar) and curcumin (Cur) self-assemble into carrier-free nanoparticles (NC NPs), which are then incorporated into thermosensitive hydrogels (NC@Gel). This approach enables efficient codelivery of both natural products, demonstrating advantages in protecting mitochondrial function and synergistically regulating key signaling pathways (Nrf2/HO-1 and NF-κB pathways) (71). At the macromaterial level, multifunctional hydrogels can be designed as explicitly assembled structures comprising a structural backbone, drug release modules, and auxiliary functional modules. For example, a chitosan-gelatin multifunctional hydrogel (CS&Gel-PA&Fe-QT&PElip) utilizes a natural polysaccharide network as its structural backbone, quercetin liposomes as the drug release module, and a protocatechuic aldehyde–iron complex as the auxiliary functional module. Through module synergy, it achieves combined antioxidant, antibacterial, and anti-inflammatory effects (72). Similarly, a two-component particulate dressing of polyacrylic acid derivatives- N-[Tris(hydroxymethyl)methyl]acrylamides loaded with madecassoside exhibits inherent antibacterial activity via ion exchange effects while continuously releasing madecassoside to exert antioxidant effects, promote M2 macrophage polarization, and stimulate collagen deposition. The entire system exhibits both wet tissue adhesion and self-healing properties (73), demonstrating the powerful ability of modular design to integrate multifunctionality.

### 6.3 Novel intelligent hydrogel systems

Moving beyond single-response characteristics, the new generation of intelligent hydrogels focuses on achieving programmable drug release and comprehensive management of the wound microenvironment (74). Multo responsive synergistic systems, such as polysaccharide hydrogels rich in curcumin and tempol dual-drug liposomes, release drugs through pH/ROS dual-response mechanisms (75). This system not only scavenges various free radicals (e.g., via curcumin), protects mitochondria, and downregulates proinflammatory factors to induce macrophage M2 polarization but also has broad-spectrum antibacterial and effective hemostatic effects. In diabetic rat models, it comprehensively promotes tissue regeneration.

The integration of bioactive carriers with natural products represents another significant trend. Natural polymer materials such as silk fibroin inherently possess excellent biocompatibility. When used as dressing substrates loaded with curcumin, they not only effectively increase fibroblast proliferation and angiogenesis but also may participate in the regulation of related signaling pathways through their structural properties, demonstrating the potential of natural materials as multifunctional delivery platforms (76).

More forward-looking explorations of plant-derived and exosome delivery systems are needed. For example, a bioadhesive hydrogel loaded with Viola yedoensis Makino-derived exosome-like nanovesicles promoted M2 macrophage polarization by downregulating NF-κB signaling, alleviating inflammation and facilitating tissue repair (77). Moreover, a composite system in which paeonol-loaded mesenchymal stem cell exosomes are encapsulated within a thermosensitive hydrogel effectively enhances human dermal fibroblast epithelial–mesenchymal transition and angiogenesis by upregulating miR-424-5p expression, significantly accelerating wound healing in diabetic mouse models; this represents a novel direction of synergistic synergy among natural products, cell-derived carriers, and smart materials (78).

In summary, through intelligent responsiveness, minimally invasive transdermal delivery, modular integration, and multi-mechanism synergy, innovative delivery systems not only overcome the inherent limitations of natural products but also enable their multitarget intervention characteristics to be precisely and efficiently executed within the complex “metabolism-senescence” microenvironment of diabetic wounds. These systems collectively form a robust technology-empowered platform, greatly advancing the translation of natural product-based therapeutic strategies for diabetic wounds into clinical reality.

## 7 Challenges, controversies, and future prospects

The field of diabetic wounds still faces multidimensional challenges arising from biological complexity, technological limitations, and clinical needs, and breakthrough solutions are urgently needed in future research.

At the level of basic research, the core challenge lies in the high complexity of the “metabolic disorders driving senescence” axis. First, senescent cells in wounds exhibit significant heterogeneity, with subpopulations showing varied responses and functions under metabolic stress, making precise clearance or regulation of specific subpopulations difficult. Second, cellular senescence in wound healing clearly plays a dual role: acute senescence induced by moderate metabolic stress can transiently promote repair, whereas excessive accumulation of senescent cells caused by chronic metabolic disorders such as persistent hyperglycemia leads to an inflammatory microenvironment and stagnation of healing (79). This requires that intervention strategies possess precise spatiotemporal resolution to distinguish and target “harmful” senescence. Furthermore, natural products, as important intervention tools, have multicomponent and multitarget characteristics. While this is advantageous for systematically regulating complex networks, it also poses significant challenges for clarifying mechanisms of action, achieving quality standardization, and ensuring controllability.

At the level of clinical translation and application, the challenges are reflected in the contradiction between individualized precision medicine and standardized protocols. The heterogeneity of diabetic wounds is rooted in differences in the systemic metabolic status of patients and is directly manifested in the local microenvironment. A recent network meta-analysis provided evidence-based support for clinical decision-making, indicating that placenta-derived tissue products demonstrate outstanding efficacy in specific types of DFUs (80), which underscores the importance of treatment on the basis of wound characteristics (such as ischemia and infection severity) rather than universal protocols. The application of technologies such as reflectance confocal microscopy for early screening of diabetic skin lesions (81) represents a trend toward early diagnosis and intervention. Although intelligent delivery systems and novel nanotherapies show immense potential for targeted delivery of natural products and synergistic treatment (82, 83), their long-term biosafety, quality control in scaled-up production, and standardized efficacy evaluation under complex mechanisms remain critical bottlenecks hindering their clinical translation.

In light of current challenges, future research should advance toward individualized, precise, and deeply integrated multidisciplinary directions. First, leveraging technologies such as single-cell sequencing, spatial transcriptomics, and metabolomics, it is essential to comprehensively map the dynamic interactions among metabolism, aging, and immunity during diabetic wound healing to lay the foundation for target discovery. Second, the development of next-generation intelligent delivery systems with spatiotemporal control capabilities is critical to achieve precise alignment between drug release and metabolic microenvironmental changes across different stages of wound healing (e.g., the inflammatory phase and proliferative phase). Finally, establishing an integrated “diagnosis–treatment–monitoring” platform, which dynamically adjusts treatment plans through real-time assessment of wound metabolic and aging biomarkers, will propel diabetic wound management into a new era of truly personalized precision medicine.

## 8 Conclusion

This review systematically elaborates on the mechanisms of impaired wound healing in diabetes and the latest advancements in therapeutic strategies. Research has indicated that cellular senescence is a central link connecting metabolic disorders with impaired wound healing and that cellular senescence is characterized by high heterogeneity and functional duality in diabetic wounds. Metabolic disturbances triggered by hyperglycemia and insulin resistance serve as the fundamental drivers initiating and amplifying local cellular senescence, whereas microenvironmental dysregulation mediated by the secretory phenotype of senescent cells constitutes the core barrier to repair. On the basis of the “metabolism–senescence axis” theory, targeting senescent cells and their microenvironment has emerged as the most promising therapeutic direction. With respect to intervention strategies, natural products and their compound formulations have unique advantages because of their multicomponent and multitarget characteristics, enabling synergistic regulation of multiple signaling pathways and exerting effects across various stages, such as antioxidant, anti-inflammatory, antibacterial, and proangiogenic effects, resulting in their distinct superiority over single-target drugs. Through precise, controllable, and efficient local drug delivery, innovative delivery systems effectively overcome the stability and bioavailability limitations of natural products, significantly increasing their therapeutic potential. In the future, interdisciplinary integration is expected to bridge breakthroughs from basic research to clinical treatment, providing more effective and safer comprehensive therapeutic solutions for diabetic wound management.
